# Obituary of Professor Omar da Rosa Santos (May 20, 1940 – November 7, 2025)

**DOI:** 10.1590/2175-8239-JBN-2026-IM002en

**Published:** 2026-05-01

**Authors:** Mauricio Younes-Ibrahim

**Affiliations:** 1 Universidade do Estado do Rio de Janeiro, Rio de Janeiro, RJ, Brazil.; 2 Pontifícia Universidade Católica do Rio de Janeiro, Rio de Janeiro, RJ, Brazil.

In November 2025, Brazilian nephrology lost one of its greatest icons, regarded by many as a “Living Legend of Brazilian Medicine.” Professor Omar da Rosa Santos was born in Rio de Janeiro in 1940, the only child of a family of Portuguese immigrants. He completed his early education at *Colégio Marista São José* and later entered the School of Medicine and Surgery (EMC) of the *Universidade Federal do Estado do Rio de Janeiro* (UNIRIO), graduating in 1964.

While still a medical student, he enthusiastically witnessed the dawn of nephrology as a specialty during the First Congress of the Brazilian Society of Nephrology (SBN), held in 1962 at the National Academy of Medicine (ANM). As if destined for it, he would later become a leading figure in both institutions. His dedication and growing interest soon made him a prominent contributor to the practice and teaching of nephrology from the earliest days of the specialty, eventually becoming one of the guardians of the institutional memory of the SBN^1^.

Professor Omar devoted himself to teaching at the very institution where he had been trained. He later served as its Director (1988–1992) and as Head of the Medical Division. As early as 1967, he was already performing peritoneal dialysis and renal biopsies. In 1968, he established the Nephrology Unit at the Central Hospital of the Brazilian Air Force in Rio de Janeiro, which later became a reference center for the training of military nephrologists.

In 1971, he became the first scholar in Brazil to obtain the title of *Livre-Docente* (habilitation) in nephrology and was appointed Full Professor of Internal Medicine in 1983. Over more than four decades of academic life, he taught thousands of undergraduate and graduate students. In recognition of his distinguished career, he was awarded the title of Emeritus Professor at UNIRIO in 2012. During that ceremony, he expressed his artistic side by creating a caricature in which the School of Medicine and Surgery was depicted as a tree, whose fruits represented its graduates, grouped according to their respective professional paths within the university (Figure 1).

**Figure 1:**
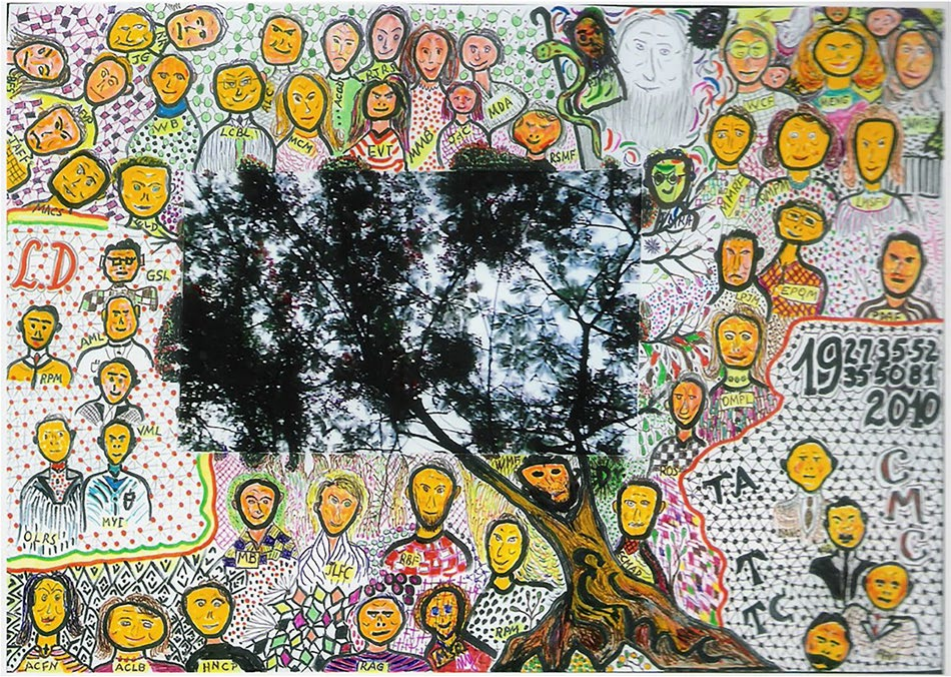
Caricature created by Professor Omar da Rosa Santos on the occasion of his appointment as Emeritus Professor at the School of Medicine and Surgery of UNIRIO.

He served as President of the Nephrology Society of Rio de Janeiro (1980–1982), founded the Nephrology Service at *Hospital do Andaraí* (1977–1992), and led the Nephrology Service of the *Hospital Universitário Gaffrée e Guinle* for decades (1966–2010). In 2000, he served as Honorary President of the Second Latin American Congress on Acute Kidney Injury organized by the Latin American Society of Nephrology and Hypertension, during which both the AKI Committee and the Latin American Renal Disaster Relief Task Force were created. He also participated in the Organizing Committee of the 2007 World Congress of the International Society of Nephrology in Rio de Janeiro, chaired by Professor Nestor Schor.

Professor Omar was a Fellow at Northwestern University (USA) and worked in numerous institutions, including the *Santa Casa da Misericórdia* in Rio de Janeiro, the Institute of Retirement and Pensions for Bank Employees, the Ministry of Education and Health, the Francisco de Castro Isolation Hospital, Antônio Pedro University Hospital (UFF), Clara Basbaum Maternity Hospital, the Health Department of the Rio de Janeiro Court of Justice, UNI-RIM Nephrology Clinic, and the Social Medicine Secretariat of INAMPS. He successfully passed 23 public competitive examinations throughout his career, a remarkable achievement that reflected his firm belief in meritocracy. He took great pride in having attained his institutional positions through these examinations.

He strongly believed in “training in service” as the cornerstone of medical education. A pioneer in implementing postgraduate *lato sensu* programs, he served as Full Professor in the nephrology specialization courses of the Carlos Chagas Foundation and the *Pontifícia Universidade Católica do Rio de Janeiro*, as well as Academic Coordinator at the Central Army Hospital. He also established highly productive medical residency programs in nephrology at *Hospital do Andaraí* and *Hospital Gaffrée e Guinle*, training generations of specialists who went on to practice throughout Brazil and Latin America.

Professor Omar was known for his distinctive and often folkloric “Prof. Omar slides,” a unique way of presenting clinical data. Despite the technical limitations of the time, he masterfully created striking visual presentations illustrating renal cellular injury, frequently using biopsy photographs with different histopathological staining techniques. He distinguished himself as an outstanding teacher, researcher, and mentor of physicians. He rarely left the microscope, patiently teaching students to analyze urinary sediment and renal pathology. Over the years, his trainees produced hundreds of scientific presentations for national and international conferences.

He was among the first to highlight the high rate of familial aggregation of end-stage renal disease among patients undergoing hemodialysis^2^. In 1988, together with Professor Annibal Nogueira, he edited the textbook *Doença dos Rins* ^3^. As one of the earliest members of the International Society of Nephrology, he collected every issue of Kidney International beginning with its inaugural edition. His humanistic approach inspired countless students and colleagues. He was always present where a physician was needed, and when he could not cure, he would provide comfort to his patients.

Professor Omar was also a pioneer in Nephrocritical Care within the Intensive Care Unit of *Hospital do Andaraí*, one of the earliest ICUs in Brazil. He dedicated particular attention to the diagnosis and treatment of acute kidney injury, exogenous intoxication^4^, infectious diseases^5^, and systemic lupus erythematosus^6^. He also compiled original renal biopsy case series across a wide range of clinical investigations, including early studies on HIV-associated nephropathy^7^. Notably, he was the first physician in Brazil to use a benzodiazepine antagonist in combination with hemoperfusion for the treatment of severe exogenous intoxication^8^.

His extensive clinical experience with acute kidney injury led to the publication of the first Brazilian textbook dedicated to the topic, *Insuficiência Renal Aguda* ^9^. At the book’s launch, he eloquently summarized the complexity of the condition:

*“Acute Kidney Injury is a nosological niche encompassing severe disorders affecting all systems and organs, arising from a broad etiological spectrum, in which prompt identification of the pathogenic cascade and appropriate therapeutic intervention are of utmost urgency.”*

Professor Omar was a true disciple of Asclepius^10^ and a member of several medical, literary, and philosophical academies. He upheld the traditions and ceremonial roles of the institutions he served with elegance and wisdom. His election as a Full Member of the National Academy of Medicine in 1989 consolidated the representation of nephrology in Brazil’s most prestigious medical institution.

He generously encouraged numerous nephrologists to pursue membership in the Academy. Through memorable speeches (Figure 2), he welcomed newly elected Full Members, including Nestor Schor, Miguel Riella, Aníbal Gil Lopes, Natalino Salgado Filho, and Mauricio Younes Ibrahim. He also received Honorary and Corresponding nephrologists, such as José A. L. Arruda (USA), Matheus Martins Prata (Portugal), Levi Guerra (Portugal), and Valdebrando Mendonça Lemos (Brazil).

**Figure 2:**
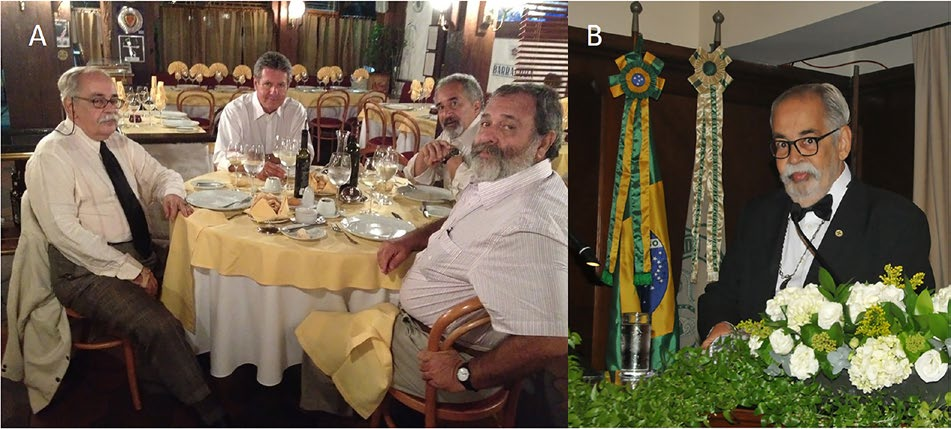
(A) Academics Nestor Schor, Miguel Riella, and Mauricio Younes Ibrahim received at the ANM by Omar da Rosa Santos; (B) Omar da Rosa Santos speaking at the ANM.

At the most recent international event organized jointly by SONERJ and the Mayo Clinic in 2024, two legendary Emeritus Professors of nephrology met: Professor Omar da Rosa Santos and Professor Richard Glassock. Through their persistence and dedication, both trained multiple generations of nephrologists across the Americas (Figure 3).

**Figure 3:**
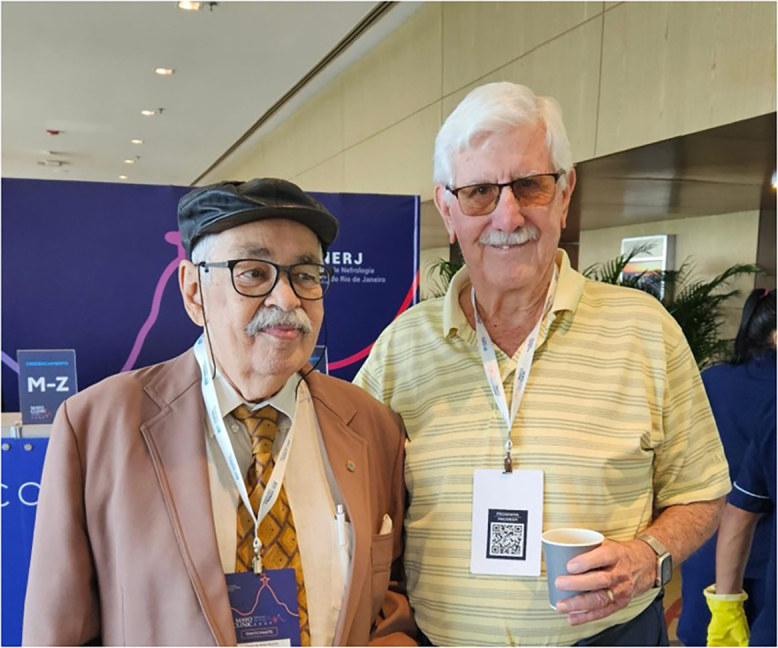
Professors Omar da Rosa Santos and Richard Glassock, in Rio de Janeiro, in 2024.

On World Kidney Day in 2025, friends and disciples honored Professor Omar by unveiling his bust at the Medical Memory Center of the National Academy of Medicine. The ceremony included remarks by the President of the Brazilian Society of Nephrology, José Moura Neto. On that occasion, one of the first Kolff-Brigham artificial kidneys in Latin America was also incorporated into the museum’s collection.

Beyond his remarkable academic legacy, Professor Omar leaves behind an enduring example of kindness, serenity, cordiality, and moderation. He also leaves a loving family: his devoted wife, Mrs. Otília Madalena Lupi Santos, their three children—Md. Omar Lupi da Rosa Santos, Md. Otília Helena Lupi da Rosa Santos, and District Attorney Olímpia Maria Lupi Santos Coelho—and six grandchildren.

## Data Availability

No new data were generated or analyzed in this study.
