# Supplementary material for: Obituary of Professor Omar da Rosa Santos (May 20, 1940 – November 7, 2025)
Source: J Bras Nefrol. 2026 May 1;48(2):e2026IM002. doi: 10.1590/2175-8239-JBN-2026-IM002en (PMC13159569; doi:10.1590/2175-8239-JBN-2026-IM002en)
Supplement: Translation

# Translation: Obituário do Professor Omar da Rosa Santos (20 de maio de 1940 – 7 de novembro de 2025)

**DOI:** 10.1590/2175-8239-JBN-2026-IM002pt

**Authors:** Mauricio Younes-Ibrahim

**Affiliations:** 1 Universidade do Estado do Rio de Janeiro, Rio de Janeiro, RJ, Brasil.; 2 Pontifícia Universidade Católica do Rio de Janeiro, Rio de Janeiro, RJ, Brasil.

Em novembro de 2025, a nefrologia brasileira perdeu um de seus grandes ícones, considerado por muitos como “Lenda Viva da Medicina Brasileira”. O Prof. Omar da Rosa Santos nasceu no Rio de Janeiro, em 1940, filho único de uma família de imigrantes portugueses. Concluiu seus estudos fundamentais no Colégio Marista São José, de onde saiu para a Escola de Medicina e Cirurgia (EMC) da Universidade Federal do Estado do Rio de Janeiro (UNIRIO), graduando-se em 1964. Ainda aluno, assistiu, entusiasmado, ao alvorecer da especialidade no Primeiro Congresso da Sociedade Brasileira de Nefrologia (SBN), ocorrido em 1962, nas dependências da Academia Nacional de Medicina (ANM). Predestinado, tornou-se um grande expoente em ambas, SBN e ANM. Sua dedicação e seu interesse crescente o transformaram em um protagonista de destaque na prática e no ensino da Nefrologia desde os primórdios da especialidade, tornando-se um dos guardiões da memória da SBN^1^.

Dedicou-se ao magistério na Escola que o formou, foi seu Diretor (1988–1992) e Chefe da Divisão Médica. Em 1967, já realizava diálise peritoneal e biópsias renais. No ano seguinte, estabeleceu a Unidade de Nefrologia do Hospital Central da Aeronáutica-RJ, que posteriormente se tornou um centro de referência na formação de nefrologistas militares. Em 1971, foi o primeiro Livre-Docente em nefrologia do Brasil e, em 1983, Professor Titular de Clínica Médica. Exerceu o magistério por mais de 40 anos, teve milhares de alunos de graduação e pós-graduação, e culminou recebendo solenemente o título de Professor Emérito da UNIRIO em 2012. Nessa celebração, deu vazão à sua veia artística e confeccionou uma caricatura na qual a EMC simbolizava uma árvore, cujos egressos estavam representados como frutos, distribuídos em diferentes cachos, agrupados pelas respectivas habilitações naquela universidade (Figura 1).

**Figura 1:** Caricatura criada pelo Prof. Omar da Rosa Santos por ocasião da concessão do título de Professor Emérito na Escola de Medicina e Cirurgia da UNIRIO.

Foi Presidente da Sociedade de Nefrologia do Rio de Janeiro (1980–1982), fundou o Serviço de Nefrologia do Hospital do Andaraí (1977–1992) e, por décadas, chefiou o Serviço de Nefrologia do Hospital Universitário Gaffrée e Guinle (1966–2010). Atuou como Presidente de Honra do II Congresso Latino-Americano de IRA, da Sociedade Latino-Americana de Nefrologia e Hipertensão, no ano de 2000, ocasião em que foram criados o Comitê de IRA da SLANH e a *Renal Disaster Relief Task Force* latino-americana. Também integrou a Comissão Organizadora do *World Congress* – *International Society of Nephrology (ISN)*, em 2007, no Rio de Janeiro, presidido pelo Prof. Nestor Schor. Foi *Fellow* na *Northwestern University* (EUA), atuou na Santa Casa da Misericórdia-RJ, no Instituto de Aposentadoria e Pensões dos Bancários (IAPB), no Ministério da Educação e da Saúde, no Hospital de Isolamento Francisco de Castro, no Hospital Universitário Antônio Pedro-UFF, na Maternidade Clara Basbaum, no Departamento de Saúde do Tribunal de Justiça-RJ, na Clínica UNI-RIM Nefrologia e na Secretaria de Medicina Social-INAMPS. Foi aprovado em 23 concursos públicos, era defensor da meritocracia e orgulhava-se dos cargos institucionais alcançados por meio desses concursos.

Acreditava no “treinamento em serviço” como ferramenta magna da formação médica, foi pioneiro na implantação da pós-graduação *lato sensu*, Professor Titular dos cursos de especialização em nefrologia da Fundação Carlos Chagas e da Pontifícia Universidade Católica do Rio de Janeiro, bem como Coordenador Acadêmico do Hospital Central do Exército. Implantou as fecundas Residências Médicas em Nefrologia no Hospital do Andaraí e no Hospital Gaffrée e Guinle, formando um contingente de discípulos especialistas, distribuídos no Brasil e na América Latina.

Originalmente, elaborava os folclóricos “slides do Prof. Omar”, uma maneira peculiar de apresentar seus dados. Com todas as dificuldades de elaboração das imagens da época, traduzia magistralmente belas projeções dos insultos celulares renais em fotografias de biópsias com diferentes colorações histopatológicas. Destacou-se exemplarmente como professor, pesquisador e formador de médicos. Não se afastava dos microscópios, ensinando pacientemente a análise do sedimento urinário e da patologia renal. Ao longo dos anos, seus alunos produziram centenas de temas livres para eventos da especialidade no Brasil e no exterior. Foi um dos primeiros a destacar a elevada taxa de agregação familiar da doença renal em estágio terminal entre pacientes em hemodiálise^2^. Em 1988, com o Prof. Annibal Nogueira, editou o livro-texto “Doença dos Rins”^3^. Como um dos primeiros sócios da ISN, colecionou o *Kidney International* desde o primeiro volume. Seu exemplo de humanismo inspirou homens, mulheres e almas; esteve presente onde o médico deveria estar e, quando não curou, confortou seus pacientes.

Foi um pioneiro do Nefrointensivismo no Serviço de Terapia Intensiva do Hospital do Andaraí, uma das primeiras UTIs do Brasil. Dedicou-se especialmente ao diagnóstico e tratamento de IRA, intoxicações exógenas^4^, doenças infecciosas^5^ e lúpus eritematoso sistêmico^6^. Reuniu casuísticas originais de biópsias renais em uma série heterogênea de investigações clínicas, incluindo os estudos iniciais da nefropatia do HIV^7^. Foi o primeiro a utilizar antagonista benzodiazepínico no Brasil^8^, associado à hemoperfusão, para o tratamento de intoxicações exógenas. Possuía uma vasta experiência clínica em IRA, que resultou no primeiro livro-texto brasileiro sobre o tema, “Insuficiência Renal Aguda”^9^. No lançamento desta obra, sentenciou com erudição:

*“A Insuficiência Renal Aguda é um nicho nosológico que acomoda agravos pungentes a todos os sistemas e aparelhos, nascidos do mais amplo leque etiológico, nos quais a presteza na identificação da cascata morbígena e a propriedade nos elos terapêuticos afiguram-se urgentes”.*

Prof. Omar era um verdadeiro discípulo de Asclépio^10^, que integrava diferentes academias médicas, literárias e filosóficas. Exercia com elegância e sabedoria as tradições e liturgias dos diversos cargos que ocupou. Sua eleição para Membro Titular na ANM, em 1989, consolidou a representação da especialidade na Casa Maior da Medicina Brasileira. Generosamente, estimulou vários nefrologistas a se candidatarem ao sodalício. Com seus memoráveis discursos (Figura 2), recepcionou os seguintes nefrologistas eleitos Acadêmicos Titulares: Nestor Schor, Miguel Riella, Anibal Gil Lopes, Natalino Salgado Filho e Mauricio Younes Ibrahim. Da mesma forma, recebeu Nefrologistas Honorários e Correspondentes: José A. L. Arruda (EUA), Matheus Martins Prata (Portugal), Levi Guerra (Portugal) e Valdebrando Mendonça Lemos (Brasil).

**Figura 2:** (A) Acadêmicos Nestor Schor, Miguel Riella e Mauricio Younes Ibrahim recebidos na ANM por Omar da Rosa Santos; (B) Omar da Rosa Santos discursando na ANM.

No último evento internacional realizado pela SONERJ/Mayo Clinic, em 2024, ocorreu o encontro de dois lendários Professores Eméritos de Nefrologia, Prof. Omar da Rosa Santos e Prof. Richard Glassock, que, com suas pertinácias e dedicações, foram responsáveis pela formação de diversas gerações de nefrologistas no continente americano (Figura 3).

**Figura 3:** Profs. Omar da Rosa Santos e Richard Glassock, no Rio de Janeiro, em 2024.

No Dia Mundial do Rim em 2025, seus amigos e discípulos o homenagearam inaugurando seu busto no Centro de Memória Médica da ANM, cerimônia que contou com a saudação do Presidente da SBN, José Moura Neto. Nessa ocasião, um dos primeiros rins de Kolff-Brigham na América Latina foi integrado ao museu da ANM.

Ao lado do grande legado acadêmico, o Prof. Omar deixa o exemplo de bondade, serenidade, cordialidade e temperança, além de uma bela família formada com sua amada esposa, Sra. Otília Madalena Lupi Santos, seus três filhos — o médico Omar Lupi da Rosa Santos, a médica Otília Helena Lupi da Rosa Santos e a promotora de justiça Olímpia Maria Lupi Santos Coelho — e seis netos.

## Data Availability

Nenhum dado novo foi gerado ou analisado neste estudo.
